# Interplay of Immunometabolism and Epithelial–Mesenchymal Transition in the Tumor Microenvironment

**DOI:** 10.3390/ijms22189878

**Published:** 2021-09-13

**Authors:** Ming-Yu Chou, Muh-Hwa Yang

**Affiliations:** 1 Department of Medicine, National Yang Ming Chiao Tung University, Taipei 11221, Taiwan; luemi0517@gmail.com; 2 Institute of Clinical Medicine, National Yang Ming Chiao Tung University, Taipei 11221, Taiwan; 3 Division of Medical Oncology, Taipei Veterans General Hospital, Taipei 11217, Taiwan

**Keywords:** immunometabolism, epithelial–mesenchymal transition, tumor microenvironment

## Abstract

Epithelial–mesenchymal transition (EMT) and metabolic reprogramming in cancer cells are the key hallmarks of tumor metastasis. Since the relationship between the two has been well studied, researchers have gained increasing interest in the interplay of cancer cell EMT and immune metabolic changes. Whether the mutual influences between them could provide novel explanations for immune surveillance during metastasis is worth understanding. Here, we review the role of immunometabolism in the regulatory loop between tumor-infiltrating immune cells and EMT. We also discuss the challenges and perspectives of targeting immunometabolism in cancer treatment.

## 1. Introduction

Metastasis is the primary cause of cancer-related mortality, which can occur early through parallel progression along with the primary tumor or late after linear tumor progression [1]. Being recognized as a major determinant of the metastatic event, epithelial-mesenchymal transition (EMT) is a reversible dynamic process in which stationary epithelial cancer cells lose their intercellular adherence, trans-differentiate into invasive mesenchymal-like cells, and initiate tumor metastasis [2,3]. During an EMT, specific changes are required by the cancer cells to migrate and colonize distant organs, including changes in intrinsic tumor cell properties and the tumor microenvironment (TME), as well as those affecting the crosstalk between the two compartments mentioned above. Amongst these changes, metabolic reprogramming has been suggested as a key hallmark of cancer progression [4,5]. Cancer cells undergo an alteration in their mode of energy metabolism to fulfill the bioenergetic and biosynthetic needs for rapid cell proliferation and adaptation to the tumor microenvironment. Apart from cancer cells, evolving studies have revealed that immune cells possess distinct metabolic characteristics that influence their immunological functions in response to cancer development [6].

Compared to the extensive understanding of metabolic alterations in cancer cells during metastasis, the role of metabolic reprogramming in tumor-associated immune cells and whether the process has mutual effects with EMT are the key questions that have not been investigated in depth. Because immunotherapy has emerged as a promising oncologic treatment, it has become increasingly vital to understand the metabolic interdependence of infiltrating immune cells and cancer as much as possible. In this review, we aim to discuss the following topics: (1) the regulatory loop between tumor-infiltrated immune cells and EMT; (2) how immune-metabolic reprogramming takes part in the loop; (3) the challenges and perspectives of targeting immunometabolism as a cancer treatment.

## 2. Epithelial-Mesenchymal Transition and Functional Change of Immune Cells in Tumor Metastasis: The Mutually Regulatory Loop

**Figure 1:**
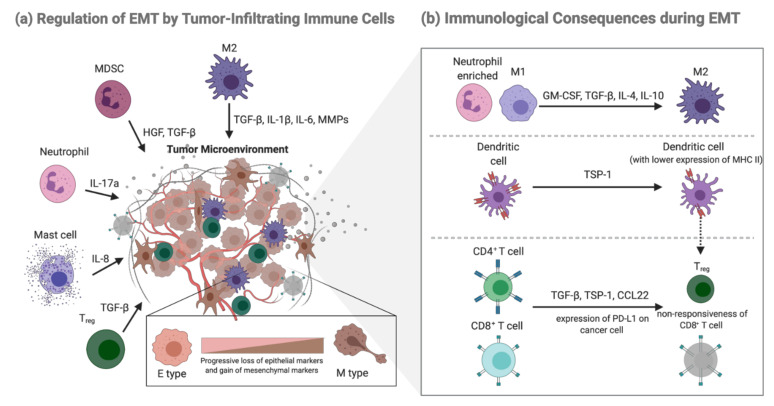
(**a**) Several immune cell types, including macrophage, MDSC, neutrophil, mast, and Treg cells, can secrete correspondent inducing factors to activate downstream signaling pathways and promote EMT. (**b**) As EMT progresses, the neutrophil-enriched TME switches to a place occupied by M2 macrophages; Dendritic cells are impaired and show lower expression of MHC II; Effective CD4+ T cells transit to immunosuppressive Treg cells, and CD8+ T cells become dull in terms of with their cytotoxic ability. Created with BioRender.com (accessed on 12 July 2021).

Alteration of the crosstalk between cancer cells undergoing EMT and tumor-infiltrating immune cells plays a dominant role in the broad spectrum of changes that occur during tumor progression. Instead of a “which came first?” question, it appears to be a mutual regulation between EMT and functional changes in immune cells (double positive feed-forward loop) during cancer development (Figure 1). This section will briefly review the possible regulatory routes between the two to provide a more comprehensive background and strengthen the causation between metabolic reprogramming (as it mainly contributes to the functional change) in immune cells and EMT, which will further be discussed in detail in the subsequent sections.

### 2.1. EMT in Cancer

EMT is a biological process that occurs during normal embryonic development, wound healing, and organ fibrosis and is also implicated in tumor metastasis [7]. This process was previously considered a binary oscillation between the full epithelial (E) and full mesenchymal (M) states; however, it has recently been viewed as a highly plastic and dynamic process, with cells lingering in an intermediate state expressing both E and M phenotypes. This hybrid E-M phenotype has been suggested to be potentially more aggressive than a complete EMT, as hybrid cells are more efficient in reaching the circulation, colonizing, and forming metastases [8,9]. While EMT has been found to contribute to invasion and metastatic dissemination, it has also been noted that mesenchymal-epithelial transition (MET)—the reversal of EMT-transits back to an epithelial state to form distant metastases [10]. For example, it was found that 62% of cases of breast cancer had increased E-cadherin at the metastatic site compared to the primary tumor, showing the possibility that tumor cells never lost E-cadherin expression or that E-cadherin expression is perturbed minimally [11]. It is likely that EMT-TFs and microRNA families that maintain an epithelial phenotype regulate MET-dependent metastatic mechanisms. While Twist1 activation (an EMT-TF) is required in promoting EMT and CTCs, turning off Twist1 at distant sites allows MET and is essential for disseminated tumor cells to proliferate and form metastases [12]; however, alternative modes of dissemination such as collective or cluster-based migration and invasion can exist, whereby cells do not need not to shed cell–cell adhesion completely while having gained the traits of migration and invasion [13]. As such, despite shifting along the EMT–MET spectrum being a salient property of primary tumor formation and metastasis, it is worth noting that the necessities of EMT and MET in tumorigenesis and metastasis are context dependent and require rigorous evaluation of the model systems employed [10]. 

Although classical EMT refers to a process in which epithelial cells lose intercellular adherence and acquire mesenchymal characteristics, a variety of other changes, including cell proliferation, apoptosis, stemness, and immunosuppression, also occur during EMT [14]. These EMT-associated changes are induced by complex regulatory networks involving transcriptional control with EMT transcription factors (EMT-TFs), including Snail, Slug, Twist1, Zeb1, and Zeb2, when activated by external signals (e.g., EGF, VEGF, PDGF, TGF-β, Wnt, and Notch) and pathological states such as hypoxia in the TME [15]. These EMT-TFs not only regulate the process as described in classical EMT, but their pleiotropic ability also allows them to be involved in other cellular functions, such as cell proliferation, apoptosis, stemness, and immunosuppression, highlighting their roles in cancer initiation, metastasis, and therapy resistance in both epithelial and non-epithelial tumors [14,16,17,18,19,20]. Apart from EMT-TFs, micro RNAs (miRNAs) and long non-coding RNAs (lncRNAs) have also been reported to regulate EMT, some of which control the expression of EMT-TFs [21,22].

These EMT-associated changes in cancer mainly occur in the tumor microenvironment, consisting of a heterogeneous population of cancer cells and a variety of resident stroma, infiltrating immune cells, secreted factors, and extracellular matrix proteins [23]. As a tumor is generally described as “a wound that never heals”, it is not hard to speculate that it is a complex interaction network in the TME that helps generate a chronic, unresolved inflammatory reaction. During a chronic inflammatory condition, TGF-β1 and hypoxia activate EMT to generate activated mesenchymal cells, notably myofibroblasts associated with tissue regeneration and fibrosis [24]; however, within the context of cancer, when chronic inflammation proceeds beyond control, these EMT programs, in an unsuccessful attempt to repair the injured tissue, turn to a vicious role and destroy epithelial homeostasis through the accumulation of the extracellular matrix in fibrosis, leading to the progression of carcinomas towards the metastatic state [25]. Apart from affecting the migratory capability of cancer cells, the immunosuppressive effect of EMT enables continuous tumor growth. The mechanism by which EMT alters the functional roles of immune cells is further discussed below.

### 2.2. EMT-Mediated Immunological Consequences

Crosstalk of the TME is composed of various interactions that dictate the outcome of tumor metastasis, while the ability of cancer cells to modulate immune responses within the tumor is one of the most studied factors. Indeed, interest in the mechanisms by which cancer cells undergoing EMT might contribute to immune evasion has increased in recent studies. A gene expression study showed that a decrease in the number of tumor-infiltrating lymphocytes (TILs), accompanied by an increased expression of immunosuppressive cytokines and inhibitory immune checkpoint molecules, is often observed in tumors with higher mesenchymal signatures [26]. In addition, a study performed on 2000 different tumors highlighted a strong correlation between EMT and markers identified with inhibited or exhausted immune responses [27]. These findings suggest that EMT is a predictive marker for immunotherapeutic outcomes.

Although much remains to be learned about the mechanisms at play, several studies performed in different types of tumors have shown that as EMT progresses, a shift from an immune profile enriched with neutrophils (a signature of inflammation) to an immune profile enriched with M2 macrophages (a signature of anti-inflammation) is observed. Numerous factors are involved in the recruitment and enrichment of M2 macrophages during EMT [28,29]. Studies have shown that cancer cells can produce various factors, including IL (interleukin)-4, IL-10, granulocyte-macrophage colony-stimulating factor (GM-CSF), and tumor growth factor (TGF)-β, which can repolarize macrophages toward an anti-inflammatory (M2) phenotype [30]. Our previous research also indicated that acetylated-Snail (a key EMT-TF) is involved in promoting tumor-associated macrophages (TAM, which is generally considered to have an M2-like phenotype) recruitment [29]. In addition to TAMs, dendritic cells (DCs) were shown to undergo differentiation into a more regulatory phenotype with low MHC class II expression after induction of snail-induced EMT cells; these impaired DCs could partly help generate Treg cells [31].

Similar to innate immune cells, immunosuppressive Tregs are induced or recruited during cancer cell EMT. Using Snail1 overexpression models of melanoma cells, it was suggested that TGF-β and thrombospondin-1 (TSP1) production apparently generated immunosuppressive Treg cells and non-responsive CD8+ T cells, resulting in enhanced tumor metastasis in various organs of the B16-F10 mouse model [31]. In HBV-positive hepatocellular carcinoma, an increase in TGF-β signaling suppresses the expression of miR-34a, resulting in enhanced production of the chemokine CCL22 and recruitment of Treg cells, promoting the development of intrahepatic venous metastasis [32]. Resistance of Cytotoxic T lymphocytes (CTLs) was also observed in the human mammary carcinoma model MCF7, which underwent EMT, following stable expression of SNAIL or after prolonged exposure to tumor necrosis factor-alpha (TNF-α) [33]. Another possible explanation for EMT-induced CTL dysfunction is the more abundant expression of PD-L1 in tumor cells. A previous study demonstrated that ZEB1, a well-known EMT activator, induces PD-L1 expression in tumor cells by relieving the miR-200 (a suppressor of EMT that targets PD-L1, a ligand for the CTL checkpoint receptor PD-1)–mediated suppression of PD-L1, resulting in the suppression of CTL function and promotion of metastasis [34]. Whether cancer cells undergoing EMT impact natural killer (NK) cells has rarely been studied, as multiple studies have shown that NK cells demonstrate little or no direct contact with cancer cells as they preferentially localize to the tumor stroma [35,36]. Consistent with this finding, emerging data suggest that circulating NK cells are potent killer cells of cancer cells compared with organ-specific or tumor-infiltrating NK cells [35]. Although there is little direct interaction between NK cells and cancer cells, an immunosuppressive TME regulated by EMT can render tumor-infiltrating NK cells with low cytotoxic activity [35].

### 2.3. Regulation of EMT by Tumor-Infiltrating Immune Cells

Not only does cancer cell EMT lead to immune evasion, emerging evidence suggest that immune cells can also regulate the process of EMT owing to their ability to produce a diverse array of EMT inducers and mediators [37,38,39].

TAMs derived from inflammatory monocytes have been shown to be potent inducers of EMT in numerous independent studies. Recruitment of TAMs through CCL2 and CCL5 results in a TME rich in TGF-β, the main inducer of EMT [40]. Consistently, analysis of primary tumors from patients with non-small lung cancer has revealed positive correlation among intra-tumoral macrophage densities, EMT markers, TGF-β levels, and tumor grade [41]. TAMs can also produce multiple cytokines (e.g., IL-1β, IL-6, matrix metalloproteinases (MMPs)), which are known to enhance TGF-β-induced EMT, subsequent invasion, and ECM degradation to promote the intravasation of cancer cells [42]. Not only do TAMs induce EMT in primary tumors, but can also secrete IL-35 in metastatic tumors to activate the JAK2–STAT6–GATA3 signaling pathway in cancer cells, which promotes MET and facilitates the colonization of cancer cells [43]. Although TAMs are involved in nearly every step of the metastatic cascade, the complexity of the interplay between TAMs and cancer cells through multiple regulatory pathways has rendered the detailed mechanisms mysterious, requiring further discovery.

In addition to TAMs, immature immunosuppressive myeloid cells named myeloid-derived suppressor cells (MDSCs), derived from abnormal differentiation of the myeloid compartment, contribute to tumor progression by involving a variety of immune suppression-dependent and -independent mechanisms [44]. In a spontaneous mouse model of melanoma, MDSCs recruited to the tumor site produced HGF and TGF-β to induce EMT, while depletion of MDSCs suppressed melanoma metastasis [44]. Intriguingly, MDSCs are also implicated in MET in cancer cells and during the formation of premetastatic niches; MDSCs reach the niche before the cancer cells and promote their seeding by secreting immunosuppressive factors, including S100A8/A9, FGF-β, IL-10, and IL-4 [45].

Compared to TAMs and MDSCs, the studies investigating the role of neutrophils in cancer EMT are relatively limited. Recently, neutrophils and mast cells have been shown to potentially induce EMT. It has been suggested that tumor-associated neutrophils (TANs) produce IL-17a, promoting EMT of gastric cancer cells through JAK2/STAT3 signaling in vivo [46]. Additionally, it was identified that the tumor-promoting effect of neutrophil extracellular traps (NETs) was closely associated with EMT in GC metastasis and promoted a pro-metastatic phenotype in human breast cancer cells [47,48]. As a participant in innate and adaptive immune responses, mast cells can contribute to the pro-tumor effect, presumably through their ability to induce angiogenesis and invasiveness. A study demonstrated that mast cells could promote an IL-8–Akt–Slug circuit that induces EMT and stemness in thyroid cancer cells [49].

In comparison with innate immune cells, there is less evidence stating that adaptive immune cells modulate the process of EMT, with Treg being the only relatively well-studied EMT modulator to date. Treg cells produce cytokines such as TGF-β, IL-6, IL-10, and TNF-α to mediate EMT. A recent study found that infiltrating Treg cells could activate Smad2/3 by secreting TGF-β1, greatly triggering EMT in hepatocellular carcinoma (HCC) [50]. The induction of EMT by TNF-α synergizing with TGF-β or other inflammatory factors has been described in human cancer cell lines in vitro [51,52]. In colorectal cancer cell lines, TNF-α and TGF-β induce EMT-like changes in an NLRP3/Snail1 axis-dependent manner, with Snail being stabilized and protected from degradation in response to TNF-α signaling, helping complete EMT and promote cancer cell migration and metastasis [53,54].

## 3. Interplay of Metabolic Reprogramming of Immune Cells and EMT

**Figure 2:**
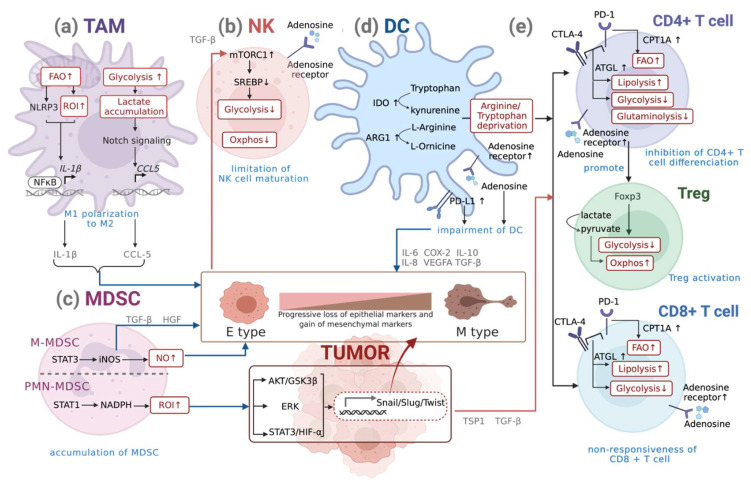
Interplay of metabolic reprogramming of immune cells and EMT. (**a**) Although expressing M2 markers, the metabolism of EMT-associated TAMs is distinct from conventional M2 polarized subset, as they prioritize glycolysis instead of OXPHOS; these TAMs also depend on FAO. (**b**) In NK cells, mTORC1 inhibition downregulates Srebp activity to decrease glycolysis and OXPHOS. (**c**) PMN-MDSCs mainly produce ROI to trigger the activation of signaling pathways such as STAT3/HIF-1α, ERK, and AKT/GSK3β, promoting several EMT-TF expressions, including Snail Slug and Twist1; M-MDSCs can also generate NO to promote EMT. (**d**) In DCs, EMT-induced high expression of IDO and ARG1 leads to depletion of arginine and tryptophan in the TME, which inhibits CD4+ proliferation and causes CD8+ T cell non-responsiveness; higher expression of PD-L1 and adenosine receptor on the cell surface impairs the function of DCs. (**e**) Via the checkpoint blockade of signaling molecules PD-1 and CTLA4 signaling, activated CD4+ and CD8+ T cells reduce glycolysis while promoting FAO and lipolysis; the downregulation of glutaminolysis alters CD4 differentiation and promotes the development of Treg cells. Being capable of converting lactate into pyruvate and supporting OXPHOS effectively, Treg cells resist lactate-mediated suppression of cell function and proliferation in the TME. Similar to NKs and DCs, higher expression of adenosine receptor on the surface of T cells suppresses the function of effector T cells. Red arrows indicate how the process of EMT can affect immunometabolism; blue arrows demonstrate how metabolic reprogramming alters immune cell function to further assist EMT. Created with BioRender.com (accessed on 18 August 2021).

From the sections above, we gained insights into how cancer cells undergoing EMT can alter the functions of immune cells and create a suppressive immune microenvironment. We also learnt about the changes in immune responses that may support the progress of EMT. Accordingly, it is increasingly vital to understand the critical factors that generate immunofunctional changes during EMT, one of which lies in the center of metabolic reprogramming (Figure 2). Alternations of metabolic pathways occurring in immune cells are crucial in performing their appropriate response, as they control downstream transcriptional and post-transcriptional events, while the dysregulation may compromise their effector functions.

### 3.1. Metabolism of Macrophages

Macrophages are versatile innate immune cells that contribute to diverse situations, including host defense, homeostasis, and pathology. Although they show phenotypic and functional diversity, macrophages mainly exhibit polarization into two distinct subsets, M1 and M2, in response to different activation stimuli. For example, through stimulation by bacterial products, such as lipopolysaccharide (LPS), or cytokines such like interferon-ɣ (IFNɣ), macrophages assume a pro-inflammatory M1 phenotype characterized by the production of inflammatory cytokines (e.g., IL-1, IL-6, IL-12, TNF), reactive nitrogen and oxygen intermediates (RNI, ROI), and microbicidal functions [55,56]. In contrast, anti-inflammatory stimuli such as IL-4, IL-13, IL-10, and glucocorticoid or immune complexes such as (IC)+LPS induce macrophages to an M2 phenotype characterized by a decreased production of inflammatory cytokines, increased production of anti-inflammatory cytokines (e.g., IL-10), and factors that mediate immunosuppression and tissue remodeling [55,56]; however, the dichotomous (M1-M2) classification used for studying macrophage activation has been based on in vitro conditions, meaning that such clear-cut phenotypes are often blurred in vivo; therefore, a transcriptome-based network analysis of macrophage activation was proposed recently, revealing that these cells integrate environmental signals in a stimulus-specific manner to induce specific functional outcomes [57]. This necessitates a multidimensional rather than a dichotomous view to describe macrophage activation states.

M1 and M2 macrophages rely on distinct metabolic pathways that promote different functions. LPS-activated macrophages show enhanced glycolysis, enabling them to generate ATP rapidly and provide biosynthetic intermediates to carry out particular effector functions such as phagocytosis and inflammatory cytokine production [58]. Apart from glycolysis, it was surprisingly found that the “redirected” TCA cycle takes part in M1 macrophage metabolism. The cycle breaks after citrate and after succinate, leading to the further promotion of fatty acid synthesis [59,60]. Here, we observed the importance of fatty acid synthesis (FAS) in M1 macrophage metabolism, as it is necessary for biosynthesis and cell growth. In fact, several studies have indicated that inflammatory stimuli could trigger an increase in fatty acid synthesis in macrophages [61,62]. In addition, the elevation of the pentose phosphate pathway (PPP) has also been shown in LPS-activated macrophages [59]. Two important outcomes of the PPP are nucleotide production (which is important for cell proliferation) and NADPH production. During infection, macrophages require NADPH to clear the infectious agent and prevent excessive tissue damage; however, with M1 macrophages showing low proliferative capacity, it remains unclear why nucleotide generation by the PPP is elevated. A possible explanation is that the nucleotides are needed to produce miRNAs and lncRNAs that are important for regulating cellular function. Amino acid metabolism is another key player in modulating the functions of M1 macrophages, with glutamine and arginine being crucial for inflammation activation, including cytokine and nitric oxide production [63,64,65]. In contrast to M1 macrophages using a redirected TCA cycle, an intact TCA cycle coupled to oxidative phosphorylation (OXPHOS) is found in M2 macrophages [60]. This allows the generation of UDP-GlcNAc intermediates necessary for the glycosylation of M2-associated receptors, such as the mannose receptor [60]. Whereas M1 macrophages prefer fatty acid synthesis, M2 macrophages rely on fatty acid oxidation (FAO), promoted by signal transducer and activator of transcription 6 (STAT6) and PPARγ-co-activator 1β (PGC1β) and inhibit inflammatory signals [66,67]. Additionally, amino acid metabolism was observed in M2 macrophages. In addition to the inflammatory involvement of arginine metabolism in the nitric oxide synthesis pathway, which is preferred in M1 macrophages, arginine flux through the arginase pathway is associated with a more tolerant immune response and is often observed in M2 phenotype macrophages [68]. The catabolism of tryptophan by macrophages may also suppress the activity of the adaptive immune system, such as inhibiting pathogen and T cell proliferation [69].

#### Metabolic Reprogramming of Macrophages during EMT

Macrophages represent a major component of the lymphoreticular infiltrates in solid tumors and play a crucial role in cancer progression [30,70]. Although experimental data suggest that tumor-associated macrophages (TAMs) are largely biased towards the M2 phenotype, evidence also indicates that TAMs are a mixed population of both M1- and M2-like macrophages, with M1 being the dominant phenotype during cancer onset, which is mainly polarized into the M2 phenotype in the late stage of cancer [43]. Metabolic plasticity and intimate crosstalk with tumor cells are essential characteristics of TAMs. TAMs respond to changes in the TME by polarizing to distinct cellular states with altered metabolic profiles. In fact, in the early inflammatory phase of cancer onset, TAMs show an M1-like phenotype and are localized in the normoxic region of the tumor, exhibiting preponderance of glycolysis, fatty acid synthesis (FAS), and PPP with a truncated tri-carboxylic acid cycle, leading to accumulation of succinate and citrate, while in the later stages of cancer, M1-like macrophages are polarized to an M2-like phenotype with greater concentrations in the hypoxic zones of the tumor, which mainly use fatty acid oxidation (FAO) and mitochondrial biogenesis [66,71].

TGF-β is the best-known inducer that activates EMT-TFs to promote EMT. Several studies have pointed out TGF-β as one of the main immunosuppressive cytokines produced by TAMs [72]. Analysis of non-small cell lung cancer (NSCLC) patients also revealed a positive correlation between intra-tumoral macrophage densities, EMT markers, TGF-β levels, and tumor grade [73]. TGF-β, an anti-inflammatory cytokine, plays an important role in polarizing TAMs to a more M2-like phenotype. Although expressing M2 markers, it was noted that the metabolism of these TAMs is distinct from that of the conventional M2 polarized subset, as they prioritize glycolysis instead of oxidative phosphorylation as a key metabolic pathway. As aerobic glycolysis is essential for EMT in cancer cells, it was reported that TAMs could compete with TME for nutrients such as glucose and undergo changes in glucose metabolism simultaneously. Recent data indicate that TAMs show high glycolytic activity, with high lactate secretion similar to the M1 phenotype [74]. Moreover, glycolytic activity seems to be essential for the M2 profile of TAMs since the inhibition of glycolysis, but not the impairment of OXPHOS or PPP, diminished the expression of M2 markers [74]. Upregulation of genes responsible for glycolysis pathways such as PDK1, PGK1, GLUT1, glucokinase (GCK), and PKM2 is seen in glycolysis-enhanced TAMs [75]. Aerobic glycolysis results in lactate accumulation in the TME, causing lactate-activated macrophages to secrete CCL5 via the Notch signaling pathway [76]. CCL5 increased cell migration, induced cancer cell EMT, and promoted aerobic glycolysis in breast cancer cells through a positive metabolic feedback loop in the co-culture system [77]. Inhibition of glycolysis in TAMs with a competitive inhibitor of hexokinase II (HK2) and 2-deoxyglucose (2DG) was sufficient to disrupt this pro-metastatic phenotype, reversing the observed increases in TAM-supported angiogenesis, extravasation, and EMT [78]. These studies indicated that TAMs, although usually phenotyped as M2-like macrophages, are metabolically similar to M1 macrophages, depending on the glycolytic metabolism used to support their functions and induce tumor metastasis.

Lipid metabolic reprogramming in cancer cells and macrophages has been shown to play an important role in cancer metastasis. Changes in arachidonic acid metabolism in cancer cells during hypoxia, including higher levels of cyclooxygenase 2 (COX-2) and its representative product prostaglandin E2 (PGE2), significantly affect EMT through β-catenin activation [79]. Studies have indicated that infiltrating TAMs and IFNγ + LPS-treated macrophages upregulate COX2 and prostaglandins, suggesting their involvement in the induction of EMT [80,81,82,83]. The significant roles of COX-2 and PGE2 in EMT induction were confirmed in a study of pharmacological COX-2 inhibitors in breast and ovarian cancer cells [84,85]. Recently, the induction of COX-2 has also been reported to be mediated by nuclear factor erythroid 2-related factor 2 (NRF2), a major mediator of oxidative stress responses [86]. NRF2 pathway has been established as a hallmark of cancer and was newly discovered acting as a “phenotypic stability factor (PSF)” for the hybrid E/M phenotype [87,88]. High expression of NRF2 observed in these hybrid cancer cells mediate their clustered migration and blunts the induction of immune response [86]. While its upregulation in cancer cells has been shown to promote chemoresistance through enhancing glutaminolysis, lactate secreted by cancer cells promoted Nrf2 activation in immune cells such as tumor-educated macrophage (TEM) [89]. It has been reported that lactate stimulation can increase intercellular ROS in macrophages, inducing macrophage M2 phenotype transformation and VEGF expression through Nrf2 mediation [90].

Cholesterol metabolic regulation has also been shown to participate in EMT. A cholesterol-lowering drug, simvastatin, was able to repolarize tumor-associated macrophages (TAM), promoting the M2-to-M1 phenotype switch via cholesterol-associated liver X receptor (LXR)/ATP-binding cassette transporter A1 (ABCA1) regulation [91]. Repolarization attenuated TGF-β, which in turn remodeled the TME and suppressed EMT [91]. In addition, accumulating evidence indicates that ceramide (a sphingolipid that can induce apoptosis) and palmitic acid (a common saturated free fatty acid that leads to lipotoxicity and apoptosis) possess the ability to modulate switching of macrophage phenotypes and provide antitumorigenic effects by altering the potential of colorectal cancer cells to undergo EMT. A recent study showed that ceramide and palmitic-acid-treated macrophages increased the expression of the M1-marker CD68 and secretion of IL-12, while the expression of the M2-marker CD163 and IL-10 secretion was attenuated [92]. Moreover, they abolished M2 macrophage-induced EMT and the migration of CRC cells. This coincided with the inhibition of SNAI1 and vimentin expression and upregulation of E-cadherin at the molecular level [92]. 

As described above, M2-like macrophages mainly undergo fatty acid oxidation (FAO). A recent study identified that FAO promotes NLRP3 inflammasome activation, which leads to increased IL-1β secretion in both mouse and human macrophages, consequently leading to enhanced EMT via hypoxia-inducible factor (HIF)-1α in HCC and pancreatic cancer cells [93,94].

### 3.2. Metabolism of DCs

In addition to macrophages, DC is another type of innate immune cell in which the metabolic requirements driving its activation and functions have been well studied. Unlike macrophages which engulf and degrade infected cells and pathogens, DCs have a relatively low phagocytic capacity [95]. Upon activation, DCs undergo morphological changes and produce cytokines and chemokines to recruit other immune cells, including T cells. Additionally, the activation state increases their ability to present protein-derived peptides on MHC molecules and various co-stimulatory ligands, including CD80 (B7-1) and CD86 (B7-2) [96].

Pro-inflammatory signals, such as TLR signaling, increase the level of PI3K-independent pathway and HIF-1α expression, which support glycolysis in DCs and turn them into a more pro-inflammatory state [97,98,99]. The process produces metabolites that can elevate PPP to enhance NADPH production or enter the TCA cycle to produce citrate needed for fatty acid synthesis, supporting cell growth and biomass accumulation [100,101]. Expression of inducible nitric oxide synthase (iNOS) generates nitric oxide (NO), a hallmark of activated DCs. Accumulation of NO downregulates OXPHOS by competing with oxygen for binding to cytochrome c, the final electron donor of the ETC [101,102]. Indeed, iNOS expression not only inhibits OXPHOS and supports glycolysis but is also required for the full maturation of DCs [101]. In contrast to pro-inflammatory DCs, tolerogenic DCs express lower MHC class II molecules and co-stimulatory ligands. Anti-inflammatory mediators including IDO, IL-10, and TGF-β have also been reported [103]. Downregulation of glycolysis and elevation of OXPHOS are seen in tolerogenic DCs, with the AMPK axis being a crucial regulator [104]. AMPK phosphorylates acetyl-CoA carboxylase (ACC) to inhibit enzyme activity, leading to increased FAO, helping replenish intracellular energy stores and support OXPHOS [105]. Upstream inflammatory signals, such as LPS and TNF-α, reduce AMPK activity and upregulate glycolysis [106,107,108].

#### Metabolic Reprogramming of DCs during EMT

Although the relationship between DC and EMT is not fully understood, a few studies point out the indirect interplay between them. Using a Snail overexpression model in melanoma, the generation of impaired DCs with low co-stimulatory molecule expression and high IDO (an immunosuppressive enzyme that acts via tryptophan deficiency) was noted, which is indirectly crucial for the induction of Treg-like CD4-CD25- cells in a cell-cell contact manner [31]. In addition to the expression of high IDO, tolerogenic DCs that express amino acid metabolism enzymes such as ARG1 and NOS_2_ also show depletion of arginine and tryptophan in the TME, which leads to inhibition of CD4+ proliferation and CD8+ T cell non-responsiveness [109].

As hypoxia is an important regulatory factor of EMT and as PD-L1 is a HIF1a target gene, it is not difficult to speculate that EMT signatures and upregulation of PD-L1 on DCs can be observed simultaneously during tumor hypoxia; however, direct causation might not exist between the two [110]. Hypoxia also creates an adenosine-rich environment and upregulates the adenosine receptor (A2bR) on human DCs, switching them to a Th2-promoting phenotype [111]. The interaction of adenosine-adenosine receptors impairs DC function. Such DCs show enhanced expression of IL-6, COX2, TGF-β, IL-10, IL-8, and VEGFA, which might further support EMT and promote tumor growth [112].

### 3.3. Metabolism of NK Cells

NK cells are cytotoxic lymphocytes that belong to the innate lymphoid cell (ILC) family and are known to defend against tumors and viral infections mainly through the production of interferon (IFN)-γ and tumor necrosis factor (TNF) [113]. NK cells do not express polymorphic germline-encoded receptors, such as TCR or BCR, nor do they require prior sensitization to carry out their functions. Upon being prompted by the engagement of receptors that recognize invariable ligands on the surface of a target cell, NK cells undergo metabolic reprogramming to support their cytotoxic activity [114].

Resting NK cells have low basal metabolic rates, utilize OXPHOS primarily, and require longer stimulation times to alter their functions [115]. Overnight incubations with IL-2 or IL-15 induce an increase in glycolysis, which feeds the TCA cycle, as well as an increase in OXPHOS to support NK cell effector function [94,116]. Glucose is the primary fuel driving enhanced glycolysis and OXPHOS in activated NK cells. Following cytokine-driven glycolysis, glucose is first converted to pyruvate and then some of the pyruvate is metabolized to lactate; however, unlike other lymphocytes that utilize pyruvate in the TCA cycle, NK cells barely feed pyruvate into the cycle. They metabolize pyruvate to cytosolic citrate via the citrate-malate shuttle (CMS) by mTORC1 signaling-driven SREBPs, which helps bypass the TCA cycle and generates both mitochondrial NADH to support OXPHOS and cytosolic acetyl-CoA to support acetylation reactions and fatty acid synthesis [94,117].

Apart from mTOR1, transcriptional factor c-Myc, which is controlled by the availability of glutamine and other amino acids, also acts as an essential metabolic regulator in NK cells; c-Myc controls the expression of glucose transporters and glycolytic enzymes required to support increased metabolism during NK cell activation [118]. As important nutrients, whether fatty acids (FAs) can fuel NK cells remains unclear. In fact, a study showed that FA administration could suppress NK cell effector functions and metabolism [119]; thus, NK cells preferentially utilize glucose metabolized by glycolysis and CMS to power effector functions.

#### Metabolic Reprogramming of NKs during EMT

As is the case for T cells, even though NK cells are present in the tumors, there is little evidence showing their capability to promote tumor progression, including induction of EMT. In fact, tumor infiltration of NK cells is primarily associated with better patient prognosis or has barely any influence. As NK cells preferentially localize to the tumor stroma, they become a major obstacle for them to mediate immunosurveillance due to limited access to cancer cells in the tumor bed [35,36,120,121]. In agreement with this finding, emerging data suggest that circulating NK cells are potent killer cells of cancer cells compared with organ-specific or tumor-infiltrating NK cells [35,122].

Despite little direct interaction existing between NK cells and cancer cells, an immunosuppressive TME regulated by EMT can render tumor-infiltrating NK cells with low cytotoxic activity. TGF-β elevation in metastatic breast cancer patients during EMT can directly alter NK cell metabolism through both mTORC1 inhibition and mTORC1-independent inhibition of mitochondrial metabolism, which downregulates Srebp activity to decrease glycolysis and OXPHOS [123,124]. In the complex TME, additional metabolites can dampen NK cell activity, such as adenosine, a key immunosuppressive metabolite that restricts the activation of cytotoxic lymphocytes. It is well known that cancer cells undergoing EMT also upregulate the expression of a 5′-nucleotidase CD73 on the surface, which converts extracellular AMP to adenosine (eADO). Excessive eADO stimulates Gs protein-coupled A2A receptors (A2AR) on NK cells, limiting their maturation [125].

Paradoxically, research has indicated that downregulation of E-cadherin (a hallmark of EMT) might enable cells to be more susceptible to NK-cell-mediated cytotoxicity, as it is a known inhibitory ligand for NK cells [105]. Consistently, acquisition of a mesenchymal-like phenotype was shown to increase the expression of NKG2D ligands, a major class of NK cell activators, rendering cells undergoing EMT more susceptible to NK cell-mediated cytotoxicity [126]. The expression of NKG2D ligands is associated with a highly active metabolism [127]. Studies have linked NKG2DL expression to active glycolysis, whereas in breast cancer, another study reported that inhibition of glycolysis increased basal NKG2DL expression [128,129,130]. Together, these studies suggest that the role of glycolysis in NKG2DL regulation is context-specific.

### 3.4. Metabolism of MDSCs

MDSCs are a heterogeneous group of immune cells derived from the myeloid lineage, possessing intense immunosuppressive activities rather than immunostimulatory properties [131]. MDSCs are classified into two major subsets: monocytic MDSCs (M-MDSCs, phenotypically similar to monocytes) and polymorphonuclear MDSCs (PMN-MDSCs, phenotypically similar to neutrophils) [132]. The detailed phenotypic characteristics of MDSCs have been described in several reviews and will not be discussed here [133,134].

Amino acid metabolism and oxidative stress, such as reactive oxygen intermediates (ROIs), are the most studied mechanisms responsible for the immunosuppressive activity of MDSCs. These effects mainly act through the following two mechanisms: (1) depletion of amino acids essential to T cells; (2) generation of oxidative stress through reactive species [131]. MDSCs deplete amino acids such as L-arginine and L-cysteine, which leads to the downregulation of the z-chain of the T cell receptor and inhibition of T cell proliferation [135]. As with macrophages and DCs, they express the inducible enzyme IDO to inhibit T cell function via tryptophan deprivation and induce the expansion of Treg cells [136,137]. By expressing NOS_2_, ARG1, and NADPH oxidase, MDSCs induce the production of RNI (e.g., NO) and ROI (e.g., H_2_O_2_) [131]. These reactive species have the same impact on T cells with amino acid deprivation, which downregulates the z-chain of TCR and IL-2 receptor signaling, inhibiting T cell activation and proliferation. M-MDSCs mainly exert their inhibitory effect by expressing high levels of STAT1 and iNOS to produce NO, while PMN-MDSCs do so through increased STAT3 and NADPH oxidase (Nox) activity, which results in the release of ROS [132,138].

While the crucial role of nitrogen and amino acid metabolism in mediating the immunosuppressive functions of MDSCs is well established, how other metabolic pathways alter their function remains relatively unknown. Although a study demonstrated that arginine metabolism and its crosstalk with carbon metabolism play a role in the maturation of MDSCs, increased FAO and fatty acid uptake can also have a regulatory role in the immunosuppressive function of tumor-infiltrating MDSCs; however, clarification of the detailed mechanisms is needed [139,140].

#### Metabolic Reprogramming of MDSCs during EMT

Not only do M-MDSC and PMN-MDSC notably use different mechanisms for immunosuppression, their ratio and distribution also vary in different kinds of human cancers. As stated above, M-MDSCs mainly exert their inhibitory effect by generating NO, while PMN-MDSCs do so by producing ROI [132]. Because of the instability of the ROI, PMN-MDSCs require close cell-to-cell contact (provided via antigen-specific interaction) to inhibit T cell function [132]. M-MDSCs produce high amounts of NO, Arg1, and immune-suppressive cytokines, which have a much longer half-life than ROI and do not require close contact with target cells to exert their effects; thus, M-MDSCs potently suppress non-specific T-cell responses and have higher suppressive activity than PMN-MDSCs [133,134].

Another study interestingly found that when it comes to EMT, multiple studies suggested that PMN-MDSC cells are involved which preferentially accumulates in the primary tumor where they become EMT “ignition agents” [141]. One possible explanation for the initiation of EMT might be the large amount of ROI produced by PMN-MDSCs. ROI triggers the activation of signal pathways such as STAT3/HIF-1α, ERK, and AKT/GSK3β and promotes the expression of several EMT-TFs, including Snail, Slug, and Twist1, thereby supporting the process of EMT [142,143]. In a spontaneous mouse model of melanoma, PMN-MDSCs recruited to tumors induced EMT by releasing TGF-β and hepatocyte growth factor (HGF). Depletion of MDSCs suppresses melanoma metastasis by inhibiting cancer cell EMT [141]. Similarly, we previously showed that colorectal cancer stem cells (CRCSCs) that have undergone EMT secrete exosomes to regulate neutrophil expansion. The signature of these neutrophils was positively correlated with the global profile of PMN-MDSCs and TANs [138]; however, in another study, it was demonstrated that a strong affinity of M-MDSCs towards tumor cells resulted in the induction of the EMT/CSC phenotype through activation of both STAT1 and STAT3 signaling pathways in tumor cells, accounting for the upregulation of EMT-related genes such as vimentin, CK14, and Twist. At the same time, PMN-MDSCs were much less involved in regulating EMT yet correlated with the proliferation signature [144]. Moreover, EMT is finely tuned by M-MDSC-mediated nitric oxide synthase (iNOS; also known as NOS_2_) in breast cancer.

### 3.5. Metabolism of T Cells

T cells are the central players in the adaptive immune response. They mature and egress from the thymus as naïve, single-positive CD4+ or CD8+ T cells. These quiescent cells then recirculate between secondary lymphoid organs while waiting for activation. Upon receiving TCR and co-stimulatory activation signals from antigen-presenting cells (APC), a network of transcriptional and metabolic programs coordinates their proliferation and differentiation into effector T cells, known as CD4+ T helper (TH) and effector CD8+ cytotoxic T lymphocytes (CTLs). As “helpers”, CD4+ T cells comprise numerous subsets (e.g., Th1, Th2, Th17, Th9, and Tfh) that serve and assist functions of other immune cells, including maturation of B cells for antibody production and activation of cytotoxic T cells and macrophages. Unlike CD4+ T cells, CD8+ T cells are cytotoxic and directly kill infected host cells by inducing apoptosis and cytokine secretion.

As stated above, when naive CD4+ and CD8+ T cells recognize their cognate antigen in the framework of co-stimulatory signaling, they become proliferative and reprogram metabolic features to support their functions [145,146,147]. For effector T cells to meet rapid proliferation, FAO, which is often used by naïve T cells, is suppressed, while aerobic glycolysis and glutaminolysis are upregulated. Mechanistically, the reprogramming process is promoted by transcription factors activated downstream of the T cell receptor (TCR) and CD28, a PI3K-AKT-mTOR pathway, such as HIF-1 (a well-known metabolic regulator during hypoxia that also takes part in T cell activation) and MYC. An increase in their transcriptional activity, in turn, upregulates enzymes that promote glycolysis (e.g., pyruvate kinase (PKM1), hexokinase 2 (HK2), glucose transporters (GLUT1) and glutaminolysis (e.g., amino acid transporters such as solute carrier proteins SLC7A5) [148,149,150]. Glucose shuttling into the PPP is also significantly increased upon CD4+ and CD8+ T cell activation, as PPP is the primary source of nucleotides (important for cell proliferation) and NADPH (required for fatty acid and plasma membrane synthesis) [148,151]. The hexosamine biosynthetic pathway (HBP) is a branch of glycolysis responsible for producing a key substrate for protein glycosylation, UDP-GlcNAc, which is critical for effector CD4+ and CD8+ T cell expansion and function [152]. Aside from glucose metabolism, activated T cells rely on amino acid metabolism to support protein and nucleotide synthesis. For example, leucine is required for mTORC1 signaling, effector function, and proper differentiation of effector CD8+ and conventional CD4+ T cells [145]. Arginine, serine, tryptophan, and cysteine supplementation also lead to improved T cell fitness and are important mediators of antitumor immune responses [69,135,153]. In addition to amino acid metabolism, fatty acid metabolism is an important regulator of T cell differentiation. De novo lipid synthesis and cholesterol uptake, which are critical for membrane synthesis, are mediated by the transcription factors sterol regulatory element-binding proteins 1 (SREBP1) and SREBP2, respectively [151,154]. Activated CD8+ T cells lacking SREBP1 and SREBP2 functionality showed dramatically decreased proliferation and antiviral activity in a mouse model. Additionally, de novo fatty acid synthesis through acetyl-CoA carboxylase (ACC) was shown to be crucial for Th17 cell differentiation while preventing the Treg cell phenotype [155]. Cholesterol uptake was also shown to enhance T-cell function. Knockout of the cholesterol esterification enzyme acetyl-CoA acetyltransferase (ACAT1) in CD8+ T cells showed improved T cell receptor signaling and increased membrane cholesterol, leading to enhanced proliferation and killing function [154]; however, a recent study demonstrated that a high cholesterol content could induce T cell dysfunction by activating the endoplasmic reticulum stress response [156]. Although cholesterol is important for effector T cell proliferation, its role in T cell metabolism remains to be elucidated.

#### Metabolic Reprogramming of T Cells during EMT

Unlike macrophages, there is relatively limited evidence suggesting that T cells modulate tumor cell phenotype directly, including induction of EMT, despite contributing to the overall tumor progression. In contrast, similar to innate immune cells, activated T cells are induced to immunosuppressive Tregs during cancer cell EMT. Using Snail1 overexpression models of melanoma cells, it was suggested that production of TGF-β and thrombospondin-1 (TSP1) appears to generate immunosuppressive CD4 + Foxp3+ T cells (Tregs) and non-responsiveness of CD8+ T cells, resulting in enhanced tumor metastasis in various organs of the B16-F10 mouse model [31]. Several studies have highlighted that a switch to the Treg phenotype indicates the preference of T cells relying on FAO and TCA cycle, which supports OXPHOS through multiple pathways [157,158,159,160,161]. Several studies have shown that when tumor cells undergo EMT, Foxp3 reprograms T cell metabolism by suppressing glycolysis, enhancing OXPHOS, and increasing nicotinamide adenine dinucleotide (NAD) oxidation [162,163]. These adaptations allow Tregs to have a metabolic advantage in a low glucose-high lactate environment (which is normally observed in the TME) through by the ability of Tregs to convert lactate into pyruvate and support OXPHOS effectively. To be more precise, as lactate accumulation has been reported to impair effector T cell function, decreasing lactate concentrations may help Treg cells resist lactate-mediated suppression of cell function and proliferation [164]. Glucose uptake and GLUT1 expression are downregulated in Treg cells compared to Teff cells in vitro. Deprivation of glucose and glutamine in media during in vitro skewing experiments has also been shown to alter CD4 differentiation and promote the development of Treg cells [165,166]; however, it is interesting that for Treg cells to exist as a highly active and long-lived phenotype, upregulation of glycolysis can optimize their function as the uptake of glucose might fuel oxidative metabolism in a manner that confers a metabolic benefit and relative advantage on Tregs in the TME [167]. Intrinsically, to allow themselves to adapt to the harsh and heterogeneous conditions in the TME, it is not surprising that Treg cells appear to have such metabolic flexibility.

In addition to promoting differentiation to Tregs, there are other ways to link EMT and T-cell-mediated immune evasion. Evidence has shown that ZEB1, a well-known EMT activator, induces PD-L1 expression in tumor cells by relieving the miR-200 (a suppressor of EMT that targets PD-L1, a ligand for the CTL checkpoint receptor PD-1)-mediated suppression of PD-L1, resulting in the suppression of CTL function and promotion of metastasis [34]. The study also indicated that NF-κB might partly regulate PD-L1 expression during EMT signaling in gastric carcinoma [168]. Interestingly, via the checkpoint blockade molecules PD-1 and CTLA4 signaling, the metabolic status of activated CD4+ and CD8+ T cells can be altered, including reducing glycolysis while promoting FAO and lipolysis [169]. Mechanistically, PD-1 promotes FAO of endogenous lipids by increasing the expression of CPT1A and inducing lipolysis, as indicated by the elevation of the lipase ATGL (the lipolysis marker glycerol and release of fatty acids) [169]. These findings suggest that immunotherapies targeting these molecules partly act by rewiring the metabolic programs of tumor-infiltrating T cells.

In addition to cell-intrinsic metabolic regulators such as PD-L1 and CTLA4, extracellular nutrients or metabolites can also alter T cell function. Human mammary cells treated with TGF-β or undergoing EMT have been shown to upregulate CD73 cell-surface expression [170]. CD73 functions as a 5′-nucleotidase, which converts extracellular AMP to adenosine (eADO). There is now a general consensus that accumulation of eADO in TME has an immunosuppressive effect that is largely mediated by excessive stimulation of Gs-protein-coupled A2A receptors (A2AR) on immune cells, including CTLs, NK cells, macrophages, and DCs [171,172].

## 4. Targeting Immunometabolism: Challenges and Perspectives

Given that metabolism plays an essential role in the generation of immune responses, many research groups are beginning to identify novel targets of immunometabolism in cancer treatment, which were well discussed and sorted in a recent review [163]. Although targeting immunometabolism has uncovered new therapeutic windows, more nuanced evaluation of metabolic demands in activated immune cells and proliferating tumor cells are still needed owing to their unfortunate similarities. To be more precise, it is a challenge to block the metabolism of tumor cells while improving the nutrient uptake of activated immune cells. Creating an environment favored by immune cells in an activated state does not always provide advantages. This environment is not conducive to the long life of the memory phenotype, which is essential for inducing a quicker, more effective adaptive response in cancer but generally prefers different metabolic pathways from the activated ones. Heterogeneity in tumors also introduces significant barriers to any novel therapy for cancer. As regions within a tumor often contain multiple genetic phenotypes, the nutrient distribution within the TME is likely distinct from one region to another. Similarly, it is not difficult to speculate that the level of metabolic perturbation varies according to tumor stage and cancer type. These results raise doubts about whether targeting immunometabolism alone could bring about the complete mobilization of the immune system against cancer. For these reasons, new approaches are required to overcome the barriers that result in a lack of therapeutic efficacy, while targeting only the most fundamental aspects of immunometabolism will be an essential issue to address.

Since the emergence of ICI drugs, novel discoveries have highlighted the roles of checkpoint receptors and their ligands in regulating cellular metabolism, both in immune and cancer cells. CTLA-4 and PD-1 receptors interfere with the signaling of CD28 co-stimulation, which acts through PI3K and Akt to increase the glycolytic rate in response to the activation required for T cells [147]. Emerging studies link checkpoint molecules with reprogramming cellular metabolism, thereby altering the function of immune cells to attenuate their antitumor ability. While PD-1 reduces glycolytic metabolism and FAS, PD-L1 expression in cancer cells promotes glycolysis via an Akt/mTOR/HIF-1α axis, while in the case of cancer harboring RAS family mutation (the mutation frequency of the Ras family in cancer is common and reaches approximately 19% [173]), the axis may be further tuned on since it was reported that RAS signaling could stabilize the expression of PD-L1 [174]. These facts suggest the synergetic effect brought about by the inhibition of both PD-1 and PD-L1 [169,175,176]. By reducing the glycolytic metabolism in cancer cells and potentially freeing up glucose in the TME, it is possible that glucose can be utilized by immune cells such as TILs, supporting the antitumor function. CTLA-4 also blocks glycolytic metabolism by inhibiting PI3K [177]. LAG-3, another negative checkpoint molecule, downregulates glycolytic and mitochondrial metabolism, potentially via elevated PTEN signaling [178]. As glycolysis is a crucial process in T cell activation, these checkpoint molecules maintain quiescence in T cells by blocking the glycolytic-related pathways, leading to an immunosuppressive TME. In addition to these inhibitory checkpoint molecules, stimulatory checkpoint molecules, such as GITR, have been shown to increase glycolysis and mitochondrial metabolism to support CD8+ T cell proliferation and effector function in vivo [179]; thus, by antagonizing stimulatory checkpoints or blocking inhibitory checkpoints is likely to restore T cell effector function by modulating cellular metabolism. Under the circumstances of understanding the limitations of simply targeting metabolism and discovering ICIs having a role in metabolic regulation, several clinical trials are underway to investigate the potential of combining metabolic interventions with conventional ICIs (Table 1). 

Clinical trials observing the effects of targeting the adenosine pathway (e.g., CD73 inhibitor, adenosine receptor inhibitor) in combination with ICIs have been sprung up in the last few years. Although most of the trials remain in the phase I stage, the results of preclinical studies have suggested targeting the adenosine pathway as an effective way to enhance the antitumor activity of ICIs [180,181,182].

Being a typical antidiabetic agent, metformin has shown its potential to convert immunotherapy-resistant patients into those showing clinical benefit. By activating AMPK, metformin decreases glycogenosis and results in the increased glucose uptake in muscle cells, leading to decreased in blood glucose levels, and subsequently decreased insulin levels. As the high numbers of insulin receptors in the cancer cells can cause tumor growth and proliferation, lowering insulin levels reduces the likelihood of malignity and prevents cancer progression [183,184]. While the effects of using metformin to target cancer cell glycolytic activity has been well investigated, metformin could directly affect infiltrating immune cells, as has been shown in the past decade. In addition to increasing CD8+ T cell recruitment, it can as also well protect them from apoptosis and exhaustion, and drive the expansion of CD8+ memory T cells [185]. It has also been shown to normalize the hypoxic TME by inhibiting the polarization of M2-TAMs via AMPK activation [186]. When combined with antiPD-1, the results indicated durable antitumor responses by preventing the presentation of PD-1+/CD8+ T-cell infiltrates after drug withdrawal [187]; however, we should acknowledge the fact that the recently reported first-generation clinical trials using metformin in combination with systemic therapy have failed to significantly improve outcomes in cancer patients [188,189]. Indeed, the synergistic effect of metformin should be reweighted when considering the apparently paradoxical association between obesity and increased anti-tumor efficacy and survival after PD-1/PD-L1 blockade (as recent evidence suggested a positive correlation between overweight and the efficacy of ICIs, the anticancer effects of metformin might be false-positive results, since metformin is a typical drug used in diabetics who often have characteristics such as overweight or obesity with metabolic disturbances) [190,191]. 

Targeting the folate pathway using pemetrexed may strengthen the anti-tumor effects by disrupting nucleotide synthesis in cancer cells. Moreover, it has been demonstrated that pemetrexed augments antitumor immunity in combination with anti-PD-L1 in mouse models, in part by enhancing effector function of CD8+ T cell through stimulating mitochondrial biogenesis with subsequent increased T cell infiltration and activation [192].

Blocking glutamine uptake is another way to starve cancer cells, meaning it is possible that less uptake would free up extracellular glutamine and in turn, reactivate the immune response. Although it remains unclear whether the blocking effect can merely be restricted in cancer cells and whether it affects immune cells, trials are still ongoing evaluating nivolumab in conjunction with CB-839, a glutaminase inhibitor, in several cancer types listed in Table 1.

Although the combined therapies of metabolic interventions with conventional immunotherapy seem promising, the failure of a recent phase III trial (named ECHO-301) of epacadostat (IDO1 inhibitor) combined with pembrolizumab has led researchers to take a deeper look at the complexity of metabolism within the TME. Multiple hypotheses have been suggested to explain these discouraging results, one of which indicates the importance of the upregulation of other compensatory pathways while blocking a targeted metabolic pathway. In this case, the inhibition of IDO1 may lead to the compensatory expression of TDO or IDO2As, two other tryptophan-degrading enzymes [193]. Another recent study reported that several IDO1 inhibitors, including epacadostat, can activate the aryl hydrocarbon receptor AhR [194]. Activation of the AhR pathway may result in the accumulation of kynurenine (kynurenine is an agonist of AHR and may participate in a positive feedback loop in AHR signaling), an immunosuppressive metabolite that induces apoptosis of effector T lymphocytes; it was also shown to increase PD-1 expression on activated T cells, turning these cells to an exhausted state [195,196]. Consistent with this finding, it may not be surprising to observe any synergetic effect of IDO1 inhibitors with anti-PD1, as blocking IDO1 would reduce PD1 expression on T cells; this would not have additional benefits if anti-PD1 fully blocks all PD1 expression. Although this might be a potential explanation for the negative result of ECHO-301, why such a phenomenon was not observed in preclinical models remains a question. Similar to IDO1 inhibitors, ARG1 inhibitors are also undergoing clinical trials in combination with ICIs. However, none of these trials reported effective results.

Above all, although targeting immunometabolism during immunotherapy sheds light on the fight against malignancy, it still appears to be challenging, as tumor cells and activated immune cells share similar utilization of metabolic pathways, meaning targeting a given pathway may concurrently disrupt the antitumor function of immune cells. Additionally, the complexity of metabolism in the changeable and heterogeneous TME makes the prediction of therapeutic efficacy even more difficult; therefore, subtle differences between the utilization of nutrients by cancer cells and immune cells should be investigated as alternate strategies to optimize the synergistic effects of the combined therapies. Likewise, understanding the interplay between different metabolic pathways may also pave the way for discovering new therapeutic targets and opportunities for modulation.

## 5. Concluding Remarks

The initiation and progression of EMT involve a robust reprogramming of the metabolism, not only in cancer cells but also in the infiltrating immune cells in the TME. Changes in immunometabolism have a central role in altering functions of immune cell which provide a logical interpretation of host immunosurveillance during EMT. In turn, these immunosuppressive activities can further promote EMT in tumor cells to aggravate cancer invasion and metastasis, indicating a malicious bidirectional regulation between the EMT program and immunometabolism. Given the pharmacological difficulties in directly targeting EMT-associated effectors such as transcription factors, highlighting the distinct immune metabolic circuits involved makes targeting EMT possible with the identification of vulnerabilities in these metabolic pathways; however, due to the similar metabolism utility levels in cancer cells and immune cells, future studies should begin to focus on the metabolic interdependence of the two and whether cancer cells can engage in metabolic crosstalk with other cells within the TME should be further investigated as well.

## Figures and Tables

**Table 1:** Ongoing clinical trials targeting immunometabolism in combination with ICIs. Bladder Urothelial Cancer (BLCA), Clear Cell Renal Cell Carcinoma (ccRCC), Colorectal Cancer (CRC), Esophagus Carcinoma (ESCA), Gastric Carcinoma (GC), Hepatocellular Carcinoma (HCC), Head and Neck Squamous Cell Carcinoma (HNSCC), Metastatic Castration Resistant Prostate Cancer (mCRPC), Microsatellite Instability/Microsatellite Stable-Colorectal Cancer (MSI/MSS-CRC), Renal Cell Carcinoma (RCC), Pancreatic Ductal Adenocarcinoma (PDAC), Triple Negative Breast Cancer (TNBC).

Pathway	Drug	Function	Combined ICI	Cancer Type	Status
Adenosine pathway	Sym024	CD73 antibody	Sym021	Solid tumors	Phase I (NCT03835949)
	AK119		AK104	Solid tumors	Phase I (NCT04572152)
	TJ004309		Atezolizumab	Solid tumors	Phase I (NCT03835949)
	NZV930		PDR001	NSCLC, TNBC, PDAC, MSS-CRC, RCC, mCRPC, Ovarian cancer	Phase I (NCT03549000)
	CPI-006		Pembrolizumab	Solid tumors, Non-Hodgkin lymphoma	Phase I (NCT0345445)
	MED19447		Duvalumab	Ovarian cancer	Phase I (NCT03267589)
	BMS-986179		Nivolumab	Solid tumors	Phase I/II (NCT02754141)
	Ciforadenant	Adenosine Receptor (A2A) antibody	Atezolizumab	RCC, Mcrpc	Phase I (NCT02655822)
	NIR178		PDR001	Solid tumors, Non-Hodgkin lymphoma	Phase II (NCT03207847)
Arginine metabolism	INCB001158	Arginase inhibitor	Pembrolizumab	NSCLC, BLCA, MSI/MSS-CRC, GC, HNSCC, Melanoma, Mesothelioma	Phase II (NCT02903914)
Folate pathway	Pemetrexed	Pyrimidine and purine synthesis inhibitor	Nivolumab	HNSCC	Phase II (NCT04107103)
	5-fluorouracil			Biliary Tract Cancer	Phase Ib/II (NCT03785873)
Glucose metabolism	Metformin	Gluconeogenesis inhibitor	Pembrolizumab	Melanoma	Phase I (NCT03311308)
				NSCLC, BLCA, MSI/NSS-CRC, GC, HNSCC, RCC, HCC, ESCA, Melanoma	Phase II (NCT04414540) (NCT04114136)
			Nivolumab	NSCLC, BLCA, MSI/NSS-CRC, GC, HNSCC, RCC, HCC, ESCA, Melanoma	Phase II (NCT03048500) (NCT03800602) (NCT04114136)
			Sintilimab	SCLC	Phase II (NCT03994744)
			Durvalumab	HNSCC	Phase I (NCT03618654)
Glutamine metabolism	CB-839	Glutaminase inhibitor	Nivolumab	NSCLC, ccRCC, Melanoma	Phase II (NCT02771626)
IDO pathway	PD-L1/IDO peptite vaccine	IDO inhibitor	Nivolumab	Melanoma	Phase II (NCT03047928)
	BMS-986205			HCC	Phase II (NCT03695250)
	Indoximod		Ipilimumab/Pembrolizumab/Nivolumab	Melanoma	Phase I/II (NCT02073123)
	KHK2455		Avelumab	BLCA	Phase I (NCT03915405)
	Epacadostat		Ipilimumab/Pembrolizumab/Nivolumab/Lirilumab	Solid tumors	Phase II (NCT03291054) (NCT03414229) (NCT03347123)

## Data Availability

Not applicable.
